# Arctic lakes show strong decadal trend in earlier spring ice-out

**DOI:** 10.1038/srep38449

**Published:** 2016-12-07

**Authors:** Tereza Šmejkalová, Mary E. Edwards, Jadunandan Dash

**Affiliations:** 1 Geography and Environment, University of Southampton, University Road, SO17 1BJ Southampton, United Kingdom; 2 College of Natural Sciences and Mathematics, University of Alaska-Fairbanks, Fairbanks, AK 99775, USA

## Abstract

The timing of the seasonal freeze-thaw cycle of arctic lakes affects ecological processes and land-atmosphere energy fluxes. We carried out detailed ice-phenology mapping of arctic lakes, based on daily surface-reflectance time series for 2000–2013 from MODIS at 250 m spatial resolution. We used over 13,300 lakes, area >1 km^2^, in five study areas distributed evenly across the circumpolar Arctic — the first such phenological dataset. All areas showed significant trends towards an earlier break-up, stronger than previously reported. The mean shift in break-up start ranged from −0.10 days/year (Northern Europe) to −1.05 days/year (central Siberia); the shift in break-up end was between −0.14 and −0.72 days/year. Finally, we explored the effect of temperature on break-up timing and compared results among study areas. The 0 °C isotherm shows the strongest relationship (r = 0.56–0.81) in all study areas. If the trend in early break-up continues, rapidly changing ice phenology will likely generate significant, arctic-wide impacts.

As a major landscape component of the Arctic, lakes play an important role in land-surface dynamics and have been shown to be sensitive indicators of climate change^1^,^2^. Many processes occurring in and around arctic lakes are influenced by the phenology of lake ice (i.e., timing of the seasonal freeze-thaw cycle). Together with the thickness of ice cover, phenology affects water temperature, thermal stratification, light penetration, nutrient supply and phytoplankton dynamics^3^. Changes in air temperature can explain up to 70% of the variance in freeze-up and break-up dates^1^,^4^,^5^,^6^, and thus ice phenology should reflect both inter-annual trends and short-term variability in regional climate^7^.

It is now established that the rate of warming at high latitudes is at least twice the global average^8^,^9^,^10^. Due to the importance of ice cover to lake ecosystems and biogeochemistry, climate feedback, and the surface energy balance, there been numerous attempts to record (or quantify) recent spatial and temporal trends in lake ice phenology in the Northern Hemispere^11^,^12^,^13^,^14^. However, the majority of earlier studies have been limited to individual large or otherwise important lakes with long series of *in-situ* observations. Only a few of these are located north of the 65°N, and they cannot be considered representative of the Arctic. Challenges to synthesis of data include non-standard protocols and terminology, absence of qualifying metadata for historical observations, varying and intermittent period of record, a lack of detailed spatial representation of arctic lakes, and changes in observation methods^15^,^16^. In addition, for the many lakes in remote arctic regions *in-situ* observations are particularly sparse, and this limits our ability to quantify the impact of warming climate on lake ice at larger spatial scales.

Air temperature, precipitation, wind conditions, location (latitude, surrounding landscape characteristics), elevation, and morphological variables such as lake size and depth are important variables governing the formation and decay of lake ice^17^,^18^. Ice formation is initiated at the moment when the heat loss at the surface of the lake exceeds the heat gained from solar radiation and convection in the lake. Lake volume determines the heat storage capacity and thus the timing of the freeze onset. Large and deep lakes generally remain ice-free longer than smaller or shallower lakes at the same latitude and altitude. The decay of ice is less dependent on the lake’s morphological parameters and is determined rather by temperature patterns and local weather conditions, as well as the volume of inflow into the lake^18^. The extent to which each of these variables determines ice phenology, and whether their influence differs geographically, has not been explored in depth at the Pan-Arctic scale, but it can be expected to contribute noise around phenological trends. Thus, large datasets are best suited to the detection of regional changes.

To date, records of ice phenology have differed in length and are relatively few, hampering detection of persistent, regional-scale trends, if they exist. In this study, we examined over 13,300 lakes over a 14-year period, a robust dataset that illustrates remarkably fast changes in arctic-wide ice phenology. This study is the first attempt to provide a detailed spatial analysis of changes in lake-ice break-up for multiple arctic regions at an inter-annual scale. In contrast to earlier studies, the current analysis considered all lakes with a surface area greater or equal to 1 km^2^ in five sampling regions across the Arctic (Fig. 1). It utilized the high spatial and temporal resolution of freely available remote sensing data. We developed a method to estimate break-up dates using time series of surface reflectance data from the Moderate Resolution Imaging Spectroradiometer (MODIS) satellite sensor and to generate a standardised estimate for over 13,300 lakes. Four key variables were extracted for each lake: 1) the date when ice is first detected, referred to as freeze-up start (FUS); 2) the date when there is no longer any detectable open water on the lake, referred to as freeze-up end (FUE); 3) the date when the first ice-free water appears, referred to as break-up start (BUS); 4) the date when the lake is completely free of ice, referred to as break-up end (BUE). However, only BUS and BUE have been analysed further due to unreliability of extracted FUS and FUE dates (see below).

Finally, the relationship between air temperature and lake-ice break-up timing was explored via a series of regional comparisons of break-up dates and air temperature-derived variables suggested in literature e.g., the annual amplitude, the date of the 0 °C isotherm^6^,^19^, fixed 30-day means^5^. While the variables governing the timing of freeze and break-up events are well defined in previous studies, our objective was to explore whether the relationships with temperature differ between study areas.

## Results

**Figure 1:**
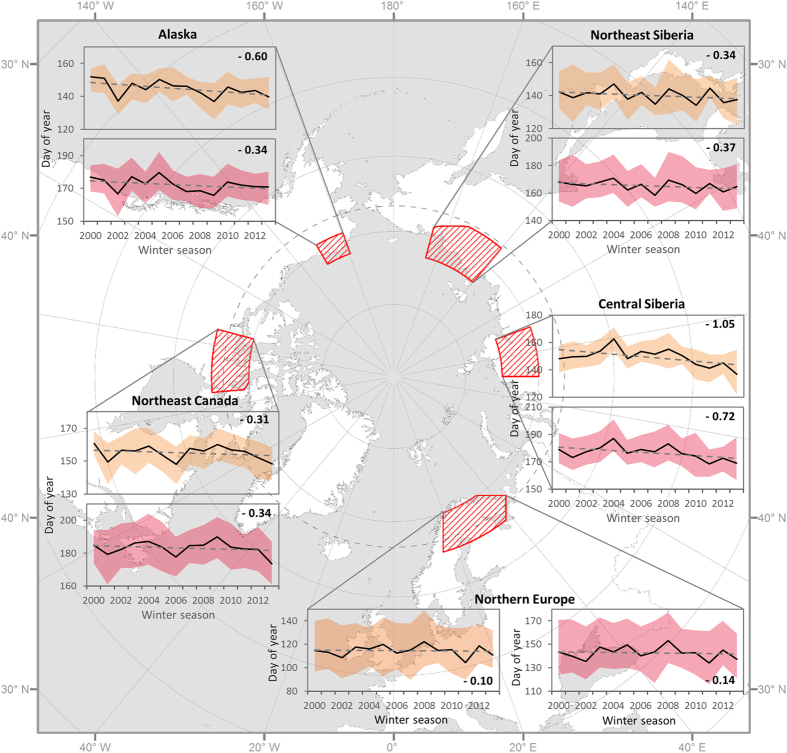
Location of study areas with average trends and dispersion of ice phenology. Break-up start (BUS, top panel/orange) and break-up end (BUE, bottom panel/pink) values are averaged for the study areas. This figure was drawn using MS Excel 2010 (http://www.microsoftstore.com) and ESRI ArcMap 10.3 (http://www.esri.com).

*In situ* observation data for 25 lakes (obtained from the Finish Environmental institute [SYKE] and the Swedish Hydrological and Meteorological Institute [SHMI]) were used to validate the satellite-derived phenological dates. There was strong agreement between *in situ* data and satellite estimates of BUE (R^2^ = 0.65, RMSE = 6.16 days, MBE = −1.38 days, n = 287). Freeze-up dates (FUE) were also estimated, but the low sun angle during the freeze-up period affected the ability of the satellite sensor to collect adequate data. This resulted in less reliable estimates of freeze-up dates and their exclusion from further analysis (R^2^ = 0.32, RMSE = 32.8 days, MBE = 16.2 days, n = 274). The earliest BUS occurred at the end of March in the southern part of the northern European study area, and the latest was in mid-June in the northern portion of the northeast Canadian study area (Fig. 1). BUE followed a similar spatial pattern three to four weeks after BUS.

Statistical analysis of the 14-year mean BUS and BUE time series showed a temporal progression to an earlier start of these events across all five sampling areas (Fig. 1). Greater variation in BUE compared to BUS is evident in the Alaskan, northeast Canadian, and central Siberian study areas (as indicated by the error bounds in Fig. 1). The central Siberian area had the strongest rate of change for BUS and BUE (slope −1.05 days/year and −0. 72 days/year respectively), whereas the northern European area had the weakest rate of change for BUS and BUE (slope −0.10 days/year and −0.14 days/year respectively). Except in the northern European study area (51% BUS, 54% BUE), the great majority (76–97% BUS, 69–95% BUE) of lakes showed a negative trend (i.e. earlier dates; Table 1). Since the statistical significance level of individual lake trends varied within study areas, presumably due to variation among lakes in such factors as water volume (see above), the overall trends and significant negative trend (p < 0.05) are discussed separately.

With the exception of the central Siberian study area, a relatively small proportion of lakes in each area showed a significant trend for the break-up period (e.g., central Siberia BUS, 33%; northeast Siberia BUS, 5%; see Table 1). Whether a trend was recognized as significant or not was strongly dependent on the completeness and length of underlying time series. For over 97% of the lakes, full 14-year BUS and BUE time series were extracted (see Methods). However, the length of record is relatively short, and this largely explains the high number of non-significant trends. In all but the Alaskan and Central Siberian study areas, more lakes showed a significant trend for BUE than for BUS (Table 1). The great majority of identified trends, whether significant or not, are negative, indicating a general pattern of earlier break-up of lake-ice. The number of positive trends (i.e. a later ice-out) corresponds to <0.1% of significant trends for all study areas except Northern Europe, which shows 17% and 29% positive trends for BUS and BUE, respectively, although absolute numbers are low (Table 1). Below, we discuss rates and spatial patterns of change by study area.

### Northern European study area

**Figure 2:**
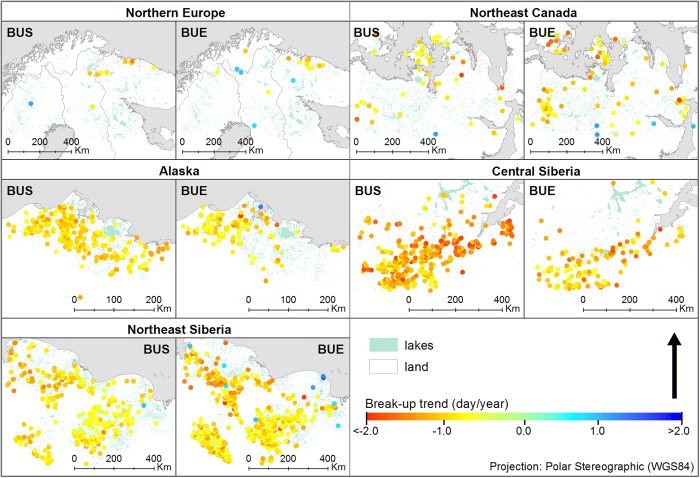
The spatial distribution and magnitude of all significant trends of breakup start and end for all study areas. Each dot represents a lake for which statistically significant trend was observed. The colour value represents the magnitude of the trend. This figure was drawn using ESRI ArcMap 10.3 (http://www.esri.com).

The trend signal is mixed for the Northern Europe study area (Fig. 2). The average overall rate of change observed for all 1,802 lakes included in the study was −0.10 and −0.14 days/year with only very few lakes (BUS 14, BUE 18) showing a significant trend. Lakes with significant negative trends (<1% of lakes) were located predominantly on the northern Kola Peninsula; here, BUS and BUE changed by on average −0.98 days and −0.89 days per year, respectively (Table 1).

### Northeast Canada study area

The overall observed rate of change for 2,994 lakes in Northeast Canada study area was stronger with mean change of −0.31 and −0.34 days per year for BUS and BUE, respectively, than observed in Northern Europe (Table 1). Significant negative trends similar in magnitude to the North European study area were observed for lakes in the glaciated terrain of the Precambrian shield of northeast Canada (Fig. 2). Lakes with significant negative trends (<3% of lakes) show a shift towards an earlier BUS, with a mean rate of −0.93 days/year. The magnitude of change in BUE was even higher with an average change of −1.05 days/year (Table 1, Fig. 2).

### Alaskan Arctic Coastal Plain study area

The 1,303 studied lakes on the Alaskan Arctic Coastal Plain show a stronger response then observed in Northeast Canada and Northern Europe, with the shift towards earlier BUS showing a mean rate of −0.60 days/year. The BUE was on average −0.34 days/year earlier (Fig. 2). Over 18% of the lakes show a significant negative trend for BUS with a mean rate −0.94 days/year; ~9% show a mean rate of −0.89 days/year for BUE (Table 1).

### Central Siberia study area

The most pronounced change in spring break-up was observed in the central Siberia study area (Fig. 2). The mean observed rate of change for 1234 studied lakes was −1.05 days/year for BUS and −0.72 days/year for BUE. Over 33% of lakes, a much higher portion than in any other study area, showed significant negative trend for BUS with mean rate of −1.40 days/year. Eleven percent showed a significant negative trend for BUE with mean rate of −1.10 days/year (Table 1).

### Northeast Siberia study area

For 6,028 studied lakes in Northeast Siberia, the largest study area, the mean observed change for BUS and BUE was −0.34 and −0.37 days/year, respectively. Many lakes (BUS 5%, BUE 8%) show significant negative trends (Fig. 2), with BUS occurring on average −0.86 days/year earlier and BUE −0.95 days/year earlier. A few significant positive trends were observed, distributed randomly, which could indicate error in the extraction process but may alternatively reflect individualistic lake behaviour, as this area has by far the highest lake density and hence number of lakes observed. If extreme and positive values are disregarded, a weak southeast to northwest gradient in the magnitude of the change can be detected, with greatest change in the northwest.

### Temperature as a controlling factor for lake ice phenology

**Figure 3:**
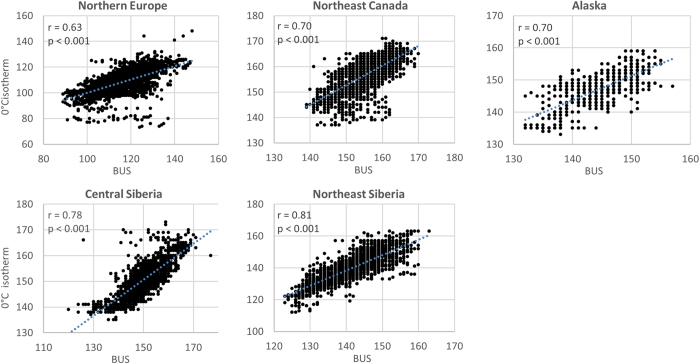
The relationship of BUS timing with the timing of the 0 °C isotherm for all study areas (axes in Julian days). Due to the spatial resolution of gridded temperature data, many lakes correspond to one grid cell. To overcome this, only the median BUS and BUE values within the grid cell were related to temperature variables. This is particularly visible for the smallest study area, Alaska, which is covered by only a few grid cells and has a low dispersion of BUS and BUE dates. This figure was drawn using MS Excel 2010 (http://www.microsoftstore.com).

We examined a range of temperature variables derived from the ERA-Interim Reanalysis 2-m air temperature dataset at a 0.7-degree spatial resolution^20^. Of these, the date of the 0 °C isotherm is most strongly related to BUS and BUE. For BUS the strongest relationship was found in the northeast Siberia study area (*r* = 0.81, p < 0.001) and the weakest for the northern European area (*r* = 0.63, p < 0.001; Fig. 3). The relationships are slightly weaker for BUE (*r* = 0.56–0.76, p < 0.001), as the end of break-up is expected to be affected by factors other than temperature alone, such as wind speed, snow cover and rainfall. These factors were excluded from further analyses due to coarse spatial resolution of the available climate data, which required downscaling of BUS/BUE for further analysis and generated noise in the regressions. Further analysis of the temperature-related variables is provided in the supplementary information.

## Discussion

While cloud cover and low illumination in winter constrain the use of optical data in the polar regions during the freeze-up period, our satellite-based method of estimating the timing of BUS and BUE provides data in good agreement with *in situ* observations during spring break-up. Overall, the results indicate a marked shortening of the spring ice-on period around the Arctic since the beginning of the new millennium (Table 1). While due to reasons highlighted above, our method is not applicable to autumn freeze-up, *in situ* observations suggest a parallel lengthening of the autumn ice-free season and shortening of the overall ice-on period^12^.

For Northern Europe, most lakes showing significant trends were located in northern Kola Peninsula. Lakes in other parts of the study area do not show any significant trend in BUS and BUE, which agrees with previous studies conducted for individual Northern European lakes^12^,^21^,^22^,^23^. This pattern is explained by regional temperature trends: unlike most of the Arctic, northwest Europe experienced stagnation in the mean annual temperature trend and even slight cooling trend in the first quartile (January-April) during the study period, except for the northern coast of Kola Peninsula, where slight warming occurred (NASA - GISS Surface Temperature Analysis).

For Northeast Canada, our results also concur with an earlier study in which the ice phenologies of six large Canadian high-latitude lakes were analysed using AVHRR surface reflectance data and which reports a mean BUE trend of −0.99 days/year over the period 1985–2004^24^.

Surdu *et al*.^25^ examined lakes in the northern part of Alaskan Arctic Coastal Plain and identified an overall negative shift in BUE date by 17.7–18.6 days over the study period 1950–2011, corresponding to −0.29 to −0.3 days/year, a rate much slower than that observed here^25^. The period 1950–1976 was considerably cooler than the period 1977–2011, and this variation in long-term temperature patterns likely masked recent rapid change^26^. In the period used in the current study, the Alaskan Arctic Coastal Plain has shown a further increase in temperatures compared with 1977–2000; the mean annual temperature increased up to +1.0 °C over the period 2000–2014 (NASA - GISS Surface Temperature Analysis, IPCC).

No published data are available for comparison for central and northeast Siberia. However, recent decadal temperature trends show a +0.5 to +2 °C increase in mean annual temperature in northern Siberia between 2000 and 2014 (more than in other study areas). This can explain the stronger response of Siberian lakes as compared to other study areas (NASA - GISS Surface Temperature Analysis, IPCC).

Identified significant break-up trends are stronger than those reported previously in literature. This is partly attributable to our use of a relatively short (14 years) and recent time period, but lake size may also be a factor, as it would be expected that small lakes (<2.5 km^2^), which are well represented in our high-resolution dataset, would be more responsive to an increase in air temperature than large lakes with greater heat storage capacity. This assumption is further supported by observation made by Arp *et al*.^27^ that bedfast ice, which is more likely to occur on small shallow lakes, leads to significantly earlier ice-out date than floating ice^27^.

The strong explanatory power of the 0 °C isotherm timing (up to 81%) agrees well with results reported in literature (North American lakes^27^,^28^,^29^,^30^). Moreover, the relationship changes only marginally among the study areas, and therefore the date of the 0 °C isotherm may usefully serve as a proxy for the timing of BUS and BUE around the circumpolar Arctic. The likely reason for the strong relationship of break-up and 0 °C isotherm is its ability to capture the variation across the extensive study areas used here, in contrast to 30-day means, which only capture local conditions.

If the trend in earlier ice-out continues, a range of implications are likely. For example, due to the large areas they occupy, lakes play an important role in shaping the local and regional climate via surface energy balance. Moreover, changes in lake-ice phenology, such as an increase in the length of the open-water season, will affect water temperatures and, in turn, within-lake biogeochemical cycles. For example, lakes that have not previously warmed above 4 °C may be driven from monomixis (one regular period of mixing, in summer) to dimixis (two mixing periods, in spring and fall), or an open-water season may be introduced to previously perennially frozen lakes^31^,^32^,^33^. Such changes would affect fundamental habitat conditions within the lake, including nutrient regimes, water column oxygenation and light availability^32^,^33^. These effects are expected to be more pronounced in small, shallow lakes with lower capacity for heat retention. Thus shifts toward shorter ice-cover duration should cause increases in primary productivity and, consequently, changes in trophic relationships within a lake^3^,^32^,^33^,^34^.

Potential direct feedbacks to climate concern the contribution to the hemispheric greenhouse gas budget. Arctic lakes in particular have been observed to be both source of and sink for carbon dioxide (CO_2_)^35^. They are also a source of methane (CH_4_), which is formed within lakes and their sediments and also via microbial processing of thawed permafrost substrates underneath thermokarst lakes^31^,^36^. Gas fluxes are likely to be climate-sensitive: for example, warming would likely enhance metabolic activity and drive higher fluxes^37^.

The observed changes in lake-ice phenology reflect the magnitude of climate change occurring in the Arctic. The trend towards earlier break-up (both BUS and BUE) in all areas is stronger than any previously identified and in some study areas exceeds one day per year. Linear extrapolation of such rates over the next several decades would bring spring break-up earlier by a whole month. Beyond potential effects on local/regional climate and lacustrine ecosystem processes, this will affect infrastructural features, such as the widespread use of lake ice for commercial and recreational winter transport routes and subsistence^32^. The data contribute to a growing range of observations that show the influence of recent warming on the arctic cryosphere^38^.

## Methods

To derive the phenological dates for the five sample areas in the Arctic an adapted temporal profile method^24^ was used. The use of surface reflectance profiles greatly reduces the volume of data necessary for processing. Fourteen years of time series of average daily near-infrared (NIR) surface reflectance for each lake were derived from the MODIS 250 m surface reflectance product (MOD09GQ, collection 5).

To identify the spatial distribution of lakes and find MODIS pixels corresponding to a lake, two data sets were used. The GLWD^39^ is widely used for regional and global studies but the majority of smaller (<10 km^2^) lakes are omitted or their area is underestimated. Recently a new and more accurate database (NALGDB) became available for the Arctic^40^. The NALGDB is based on Landsat imagery and covers the area north of 65° latitude. With an overall accuracy of 78% and 30-m resolution, it is the most complete and accurate database available for Arctic lakes. However, while small lakes are (fairly) accurately mapped, some large lakes were excluded from the version we used. Therefore, both databases were combined to provide a comprehensive dataset across lake sizes greater than 1 km^2^. Minimum lake area was set to 1 km^2^ in order to ensure that at least one pure lake pixel could be obtained from the MODIS data.

The NIR surface reflectance profiles (Band 2 of MOD09GQ) were extracted for all MODIS pixels within a lake boundary. Although low quality pixels were masked prior to extraction using the MODIS Quality Assurance (QA) flag^41^, residual extreme values caused by atmospheric conditions (cloud, haze) may still have been included, thus affecting the final mean lake reflectance profile. A method to detect and remove outliers was implemented, which used the mean absolute deviation from the median in a moving temporal window (for details see supplementary material).

Following outlier removal, mixed land/coastal pixels falling within the boundary of a specific lake were removed from the set of lake pixels. To identify mixed land water pixels subset of 1000 pure-lake reference pixels was selected from the data. Mean summer (July and August) reflectance in every year was calculated for all pixels in the subset. The 3rd quartile (390.4) of all the means was set as the open-water reflectance threshold in case residual ice was present in some reference pixels. The July-August pixel mean reflectance was then calculated for all temporal profiles of all lakes in the study for the first 5 years (2000–2004). If the means were higher than the reference threshold for more than 2 out of 5 years, the pixel was considered mixed and the profile was discarded. Due to the rugged shorelines of Arctic lakes, almost 75% of profiles were removed for some of the lakes. For approximately 40% of the original 23,234 lakes across the study areas, all the profiles were removed, meaning those lakes did not contain any pure lake pixels and were excluded from further analysis (e.g., small lakes or lakes with very rugged boundaries and/or islands). Finally, the pixel temporal profiles were averaged for each lake to give the final lake NIR surface reflectance profiles.

The extraction of FUS, FUE, BUS and BUE dates from the mean profiles was performed using TIMESAT software^42^. Features in the lower portion of the reflectance profile (i.e. FUS, BUE) are likely to be affected by erroneous bright values (cloud, haze, etc.)^24^. To overcome this, these variables were derived from an adapted curve that was fitted to the lower envelope of the surface reflectance time series (see supplementary material). On the other hand, high reflectance values (i.e. FUE, BUS), are more susceptible to dark artefacts (e.g., shadow). Therefore, these were derived from a curve fitted to the upper envelope of the time series. Due to low sun illumination, freeze-up events (FUS and FUE) do not appear as distinct features in majority of profiles, and cannot therefore be extracted with confidence (see Supplementary Material).

Finally, the extracted BUE dates were validated using *in situ* observations for 25 lakes in Northern Europe (288 observations). Agreement between *in situ* observations and estimates was measured using simple correlation, coefficient of determination (R^2^), mean absolute error (MAE) and root mean square error (RMSE).

To analyze a time series for a trend, the Theil-Sen slope approach was used^43^. The significance of trends was assessed using the rank-based, non-parametric Mann Kendall test. To correct for serial correlation, the approach proposed by Zhang *et al*. was used^44^. The trend analysis was performed using the ‘zyp’ (Zhang + Yue-Pilon trends) package in R^45^. For the calculation of the mean trend magnitude in each area, 2% of lowest and highest values have been removed to avoid skewness due to outliers.

Finally, to explore the strength and significance of the relationship between annual temperature amplitude, the date of the 0 °C isotherm, 30-day daily air temperature means and the timing of the break-up events the BUS and BUE dates were estimated using Pearson product-moment correlation *r*. The date of 0 °C isotherm was defined as the date when the daily air temperature profile, smoothed in a 31-day window, crosses the 0 °C value. The fixed-period means were calculated within a running window of 30 days, with a 10-day step from mid-February to end of July (for more information see supplementary material). The strength and the significance of relations between freeze-up/break-up dates and autumn/spring isotherm were estimated using Pearson product-moment correlation.

## Additional Information

**How to cite this article**: Šmejkalová, T. *et al*. Arctic lakes show strong decadal trend in earlier spring ice-out. *Sci. Rep.* **6**, 38449; doi: 10.1038/srep38449 (2016).

**Publisher’s note:** Springer Nature remains neutral with regard to jurisdictional claims in published maps and institutional affiliations.

## Supplementary Material

Supplementary Information

## Figures and Tables

**Table 1:** Summary of observed changes.

Study area	Mean – BUS (number of lakes)	Mean significant – BUS (number of lakes)	Mean – BUE (number of lakes)	Mean significant – BUE (number of lakes)
Northern Europe	**−0.10 (1,802)**	−0.84 (14)	**−0.14 (1,804)**	−0.52 (18)
*Negative*	−0.44 (937)	−0.98 (13)	−0.44 (982)	**−0.89 (14)**
*Positive*	0.39 (603)	1.09 (1)	0.35 (523)	0.79 (4)
*None*	0 (262)	—	0 (299)	—
Northeast Canada	−0.31 (2,994)	−0.89 (54)	−0.34 (2,995)	−0.96 (82)
*Negative*	−0.44 (2,297)	−0.93 (53)	−0.57 (2,072)	−1.05 (78)
*Positive*	0.26 (337)	1.04 (1)	0.30 (546)	1.04 (4)
*None*	0 (360)	—	0 (377)	—
Alaskan Arctic Coastal Plain	−0.60 (1,303)	−0.94 (247)	−0.34 (1,303)	−0.87 (116)
*Negative*	−0.64 (1,215)	−0.94 (247)	−0.46 (1,070)	−0.89 (115)
*Positive*	0.23 (43)	—	0.29 (172)	1.35 (1)
*None*	0 (45)	—	0 (61)	—
Central Siberia	**−1.05 (1,234)**	−1.40 (414)	**−0.72 (1,235)**	−1.10 (143)
*Negative*	−1.08 (1,202)	**−1.40 (414)**	−0.76 (1,176)	**−1.10 (143)**
*Positive*	0.29 (17)	—	0.30 (27)	—
*None*	0 (15)	—	0 (27)	—
Northeast Siberia	−0.34 (6,028)	−0.85 (312)	−0.37 (6,028)	−0.92 (531)
*Negative*	−0.44 (4,864)	**−0.86 (311)**	−0.48 (4,968)	−0.95 (522)
*Positive*	0.21 (397)	0.83 (1)	0.29 (471)	0.96 (9)
*None*	0 (767)	—	0 (589)	—

The mean BUS/BUE is the mean change (days per year) in the BUS/BUE timing calculated from all lakes within a study area. The mean significant change/trend only includes lakes where trends are significant at 95% confidence level. The mean BUS/BUE in the spring break-up in days/year differed noticeably among study areas, but the mean significant BUS/BUE trends show much lower variation. The bold print shows the largest and smallest trend among the study areas.
