# Supplementary material for: Arctic lakes show strong decadal trend in earlier spring ice-out
Source: Sci Rep. 2016 Dec 7;6:38449. doi: 10.1038/srep38449 (PMC5141450; doi:10.1038/srep38449)
Supplement: Supplementary Information [file srep38449-s1.pdf]

Supplementary information

Arctic lakes show strong decadal trend in earlier spring ice-out

Tereza Šmejkalová, Mary E. Edwards, Jadunandan Dash

Supplementary Discussion 1 – Method details

A. Data

A.1 Satellite data

The study is based on data acquired by the Moderate Resolution Imagining Spectroradiometer (MODIS) on board of the Terra satellite, specifically the daily MODIS Surface Reflectance Daily L2G Global 250m product (MOD09GQ). The level 2G-lite product MOD09GQ has been corrected for the effects of atmospheric scattering and absorption, composited on daily level, gridded into MODIS sinusoidal projection and cut into approximately 10°x10° granules[1].

The quality band of this product, however, is not populated, and the 1-km Reflectance Data State band from the MODIS Surface Reflectance Daily L2G Global 500 and 1-km product (MOD09GA) must be used instead. For the purposes of this study only the near infrared band (band 2) of MOD09GQ product was used. This part of the spectrum is most suitable for lake ice detection due to very low reflectance of open water and therefore higher contrast between water and ice. Moreover the NIR band is not as heavily affected by atmospheric conditions as the red wavelengths[2].

As with all optical sensors, MODIS imagery is affected by low illumination in polar areas during fall and winter. For this reason, data from November to February (date depending on study area) were considered unavailable. The start and end of the low illumination period changes with latitude and additional correction is needed remove affected pixels.

A.2 Lake Datasets

Several datasets have been used to create the underlying lake database for this study. Originally only two datasets were considered. The Global Lake and Wetland Database (GLWD) developed by[3] is considered the most complete global database available. It is widely used for regional and global studies but majority of small (>10km^2) lakes mainly in the Arctic is omitted or their area is underestimated. The Arctic lakes that are mapped show slight shift in their location which makes the database unsuitable to use as a mask for satellite data extraction. Recently a new and more accurate database became available do the arctic areas created by Paltan[4]. The New Arctic Lake Geodatabase (NALGDB) is based on Landsat imagery and covers area north of 65°N. With overall accuracy of 78% and 30m resolution it is the most complete and accurate database of Arctic lakes available. While small lakes are mapped very well, most of the large lakes are missing in the version that we used.

Due to drawbacks of both databases it was decided that their combination would yield the best results. As the more accurate, the NALGDB was used as a base, and only lakes not included in the NALGDB were then selected from the GLWD. Both were merged into a final dataset for each study area. The result was checked visually over Google Earth. For all study areas the final lake dataset was projected to Polar Stereographic projection as defined by the WGS84 datum with central meridian at 0° E and latitude of origin at 71°N (EPSG code 7215).

A.3 In-situ data – validation data

The availability of *in-situ* observations for lake ice phenology events is limited in arctic areas. Only two datasets were acquired, both within the north European study area. Freeze-up end (FUE) and break-up end (BUE) time series were obtained from the Finish Environmental institute (SYKE) and the Swedish Hydrological and Meteorological Institute (SHMI) for 25 lakes. Some of the time series are incomplete.

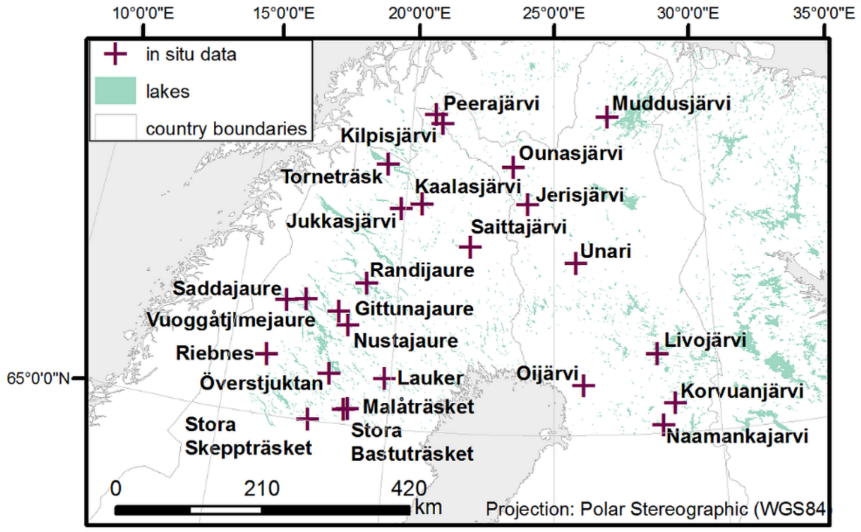

Figure S1 | Map of lakes for which in-situ observations are available. In total there are 288 observations for break-up and 275 for freeze-up (Fig. S1). This figure was drawn using MS Excel 2010 (http://www.microsoftstore.com), the location map was created using ESRI ArcMap 10.3 (http://www.esri.com) and open source coastline data from DIVA-GIS (http://www.diva-gis.org/Data). Lake boundary was created using data from new Arctic lake database (http://eprints.soton.ac.uk/id/eprint/388785).

A.4 Climatic data

Daily temperature, wind speed, precipitation and snow depth data were acquired from the ERA Interim dataset. Gridded ERA Interim is global atmospheric reanalysis dataset produced by the European Centre of Medium-range Weather Forecast (ECMWF). It covers period from 1st January 1979 till present and continues to be produced in near-real time. The data were downloaded in NetCDF format for the entire Arctic. This dataset has been selected due to its spatial resolution of 0.75 degrees, which one of the finest for global climatic datasets. Mean daily data are not available, therefore it was calculated from 6-hourly data.

A.5 Definition of studied phenological events

Four observed phenological events are: Freeze-up start (FUS) - ice is first detected, Freeze-up end (FUE) – no detectable open water on the lake, Break-up start (BUS) – first appearance of open water, Break-up end (BUE) – lake is completely ice free [2]

B. Phenology extraction

B.1 Surface reflectance profile preparation (Fig. S2)

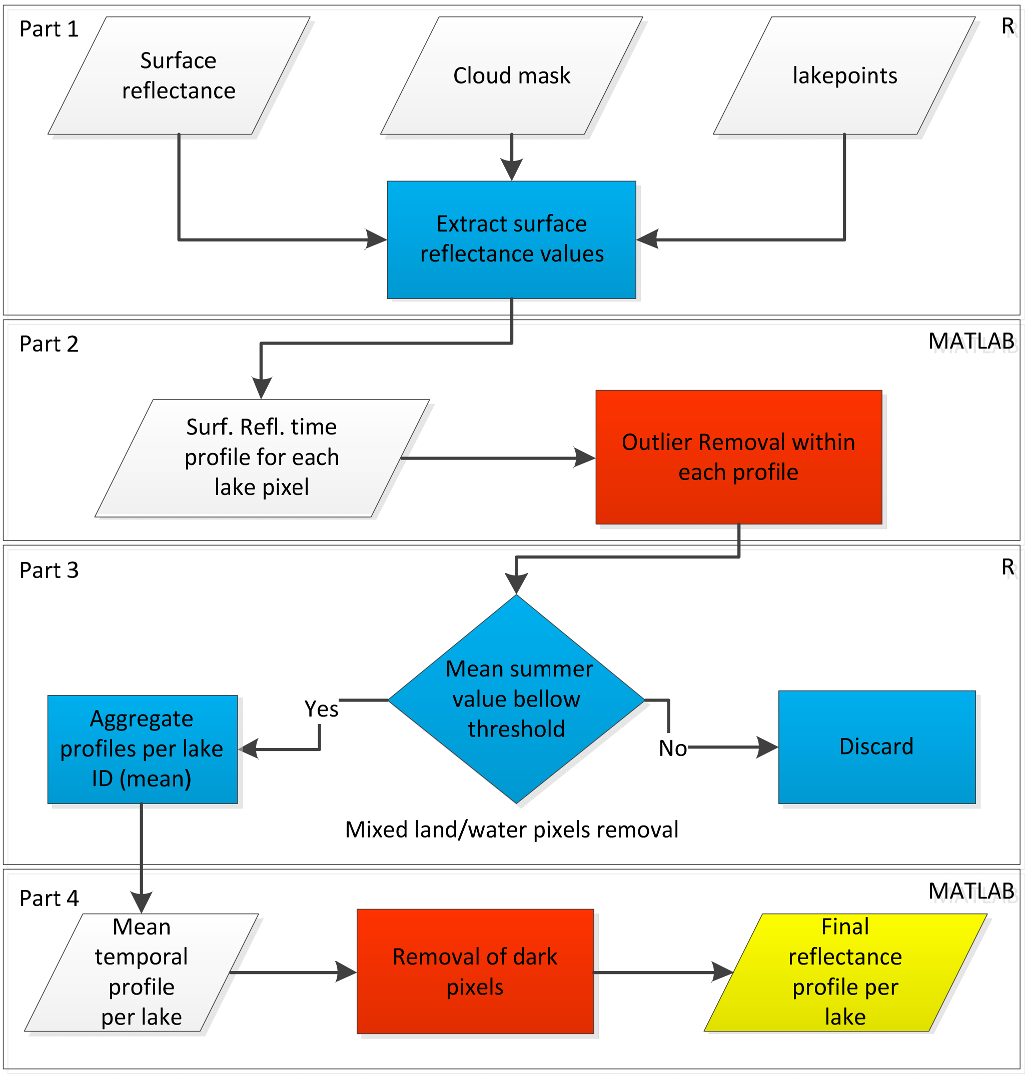

Figure S2 | Flow-chart for preparation of the surface temporal reflectance profile. The use of temporal surface reflectance profile greatly reduces the data volume. This figure was drawn using Microsoft PowerPoint 2010 (http://www.microsoftstore.com).

B.1.1 Satellite data pre-processing

Due to the significant size of the original MOD09GQ and MOD09GA tiles the data had to be downloaded and processed continuously. This was done using the Aria2 download utility and MODIS Reprojection Tool (MRT) via script developed in R. For each of the five study areas the NIR and state band were extracted, mosaicked, reprojected and resampled to 250 m. All data were converted into single band TIFF images of equal extent. In total, nearly 90 000 MOD09GQ and MOD09GA HDF tiles were processed over all study areas.

The state data were reclassified to binary cloud mask using code developed in Python based on ESRI ArcGIS functionality. The reclassification was based on a look-up table in ASCII format containing all state values (0-65535) and their corresponding class. Classes were assigned to decimal values based on the corresponding 16-bit binary number and State QA description available from [1]. Only cloud-free pixels with no cloud shadow and no cirrus cloud detected were classified as 1; all lower quality pixels were classified as 0.

B.1.2 Lake point dataset

The first step was preparation of a lake-point dataset for each study area containing coordinates at which values from the satellite data would be extracted. Each lake in the final study area polygon was assigned unique ID sorted by latitude of its centroid. It is crucial that the points correspond to the centroid of cells in the resampled imagery. The polygon dataset was then converted to a raster aligned with the pre-processed satellite data and with a corresponding cell size (250 m), and the unique lake ID was used as the value field. The raster was then converted to a point dataset where each point represents the centre of a raster cell. Apart from the unique lake ID, each point was assigned a unique point ID and set of coordinates in Polar Stereographic projection. The point dataset was then exported into an ASCII format.

B.1.3 Profile extraction (Part 1)

The surface reflectance values were extracted using code developed in R and using the R raster package. The extraction was done for one year at a time. The inputs were surface reflectance daily images and corresponding cloud mask data, both sorted by day of year, and the lake point dataset. The output is in the form of ASCII file, where each row represents daily reflectance values for one input point (Fig S3). As mentioned in the *Data* section, imagery from winter period from 1st December to 8th – 26th February (depending on location) were omitted from processing completely due to low illumination. Moreover, in several years a number of dates is not available due to MODIS technical issues. In such cases and in the case of cloud cover, a No Data value (NaN) was assigned. All profiles show 75 – 88% of missing data. Although MODIS data are only available from 24th February 2000, the 1st January 2000 is considered as first day of study (DOS).

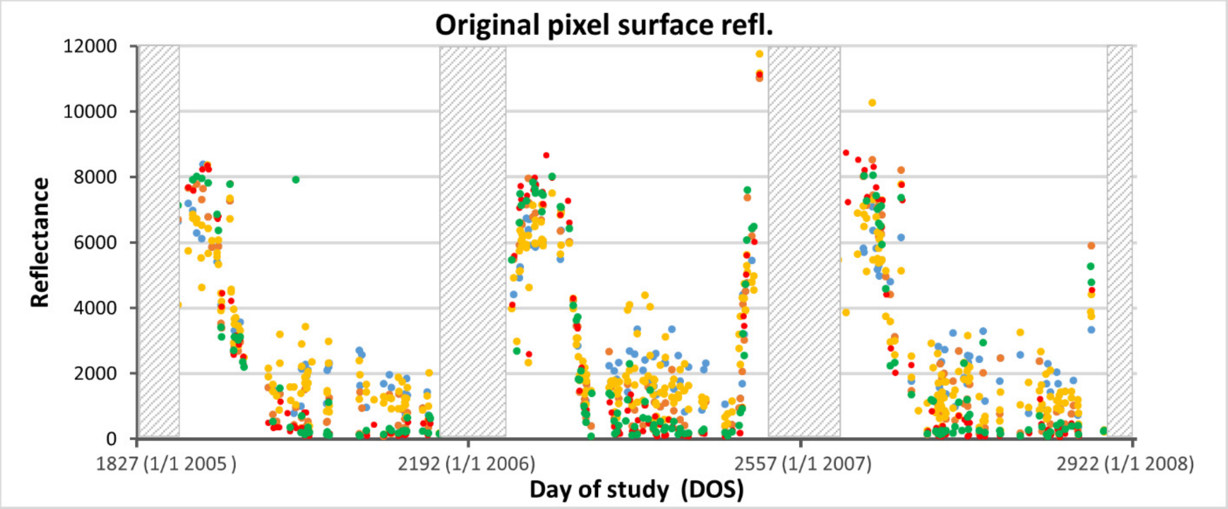

Figure S3 | Example 3-year subset (2005-2007) of extracted daily surface reflectance values for Stor-Bastuträsket lake in central Sweden. The lake covered by 8 pixels (output of Part 1) – each pixel denoted by different colour. Grey rectangles show periods for which data were not processed due to low sun illumination affecting the reflectance values. This figure was drawn using MS Excel 2010 (http://www.microsoftstore.com).

B.1.4 Outlier removal (Part 2)

Although low quality pixels were masked prior to value extraction, residual outliers still may be present within the profile. These values would affect the final mean lake profile, and therefore a method to detect and remove them was devised in MATLAB. Leys et al. (2013)[5] discussed the use of mean absolute deviation from median as the most robust dispersion measure in the presence of extreme values and, therefore, ideal for their detection. Unlike the frequently used

standard deviation from mean, MAD is not so strongly affected by extreme values. Moreover, it is applicable even to very small sample sizes. Median absolute deviation (MAD) was calculated within each window. MAD is defined as follows:

$$MAD = b * M_i(|x_i - M_j(x_j)|) \qquad \text{(Eq. S1)}$$

Where b is a constant defining the data distribution, here b = 1.4826 for normal distribution. $M_j(x_j)$ is the median of n original values and M_i is the median of absolute deviations from the sample median. The MAD was calculated in an 11-day moving window. A conservative threshold of 3*MAD from the window median was selected to define outliers. If the central value of the sample exceeds this threshold it is assigned the NoData value, but only under the condition that a minimum of 5 non-missing values are present in the window.

B.1.5 Removal of mixed pixel profiles (Part 3)

Many pixels for which the reflectance profile was extracted correspond to mixed land/water cover. The reflectance of land is much higher than that of open water in summer and, depending on snow cower, it can be lower than ice reflectance in winter. Therefore, mixed pixels would introduce errors into the final mean lake profile and affect the accuracy of feature extraction. The effect of mixed reflectance can be seen in Fig. S4 (violet and yellow time series).

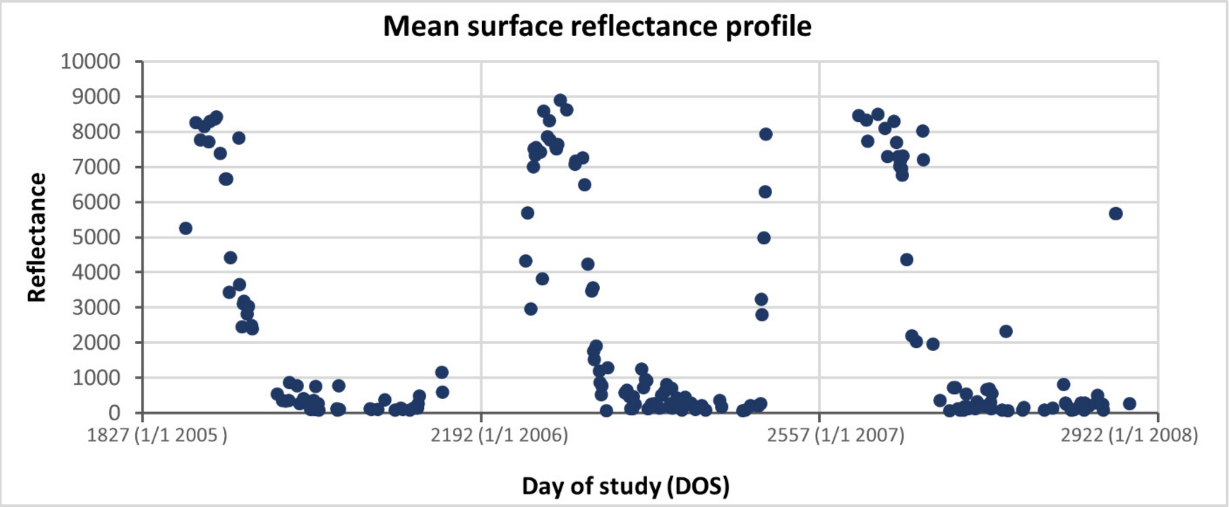

Figure S4 | Example 3-year subset (2005-2007) of mean lake surface reflectance profile for Stor-Bastuträsket lake in central Sweden. This figure was drawn using MS Excel 2010 (http://www.microsoftstore.com).

The first step was to define an open water reflectance threshold. One thousand reference open water points were selected from the dataset over high resolution imagery. For each of these points mean reflectance for two summer months (July and August) was calculated over 5 years. July and August were selected as months for which the lakes are most likely to be ice-free. In the case some of residual ice on some lakes, the 3rd quartile (390.4) of all the means was then set as the open water reflectance threshold.

The next step was to identify and remove the mixed pixel profiles. For all the points in the original dataset summer means for 5 years were calculated, as for the reference points previously. All means were then compared with the threshold. If the summer means were higher than the threshold for more than 2 out of 5 years, the profile was discarded. Due to ragged coastlines of Arctic lakes almost 75% of profiles were removed. For approximately 40% of

lakes, depending on the study area, all the profiles were removed eliminating them completely from the analysis.

In the final step, all pixel profiles per lake were averaged to produce mean surface reflectance profile per lake (Fig. S4). The whole process was done using an algorithm written in R.

B.1.6 Removal of 'dark pixels' (Part 4)

In fall, the effect of low sun illumination on the surface reflectance starts to be visible from mid-October, depending on the study area (16th October for the central Siberian area, 29th October for the Alaskan area, and 3rd November for the areas in Northern Europe and northeast Canada). The affected portion of the image gradually increases until the end of November and decreases again to a clear image 8th – 26th February, depending on the study area. The reflectance values of affected 'dark' pixels are close to 0; however, the state data do not classify these pixels as low quality. For spring, all affected images can be removed from analysis because the start of break-up period occurs considerably later. On the other hand, the freeze-up period in the Arctic occurs when the sun radiation decreases during October and November. Therefore, it is crucial for estimation of FUS and FUE to preserve as many valid observations for November as possible.

In this period a majority of profiles show rise in reflectance values suggesting the start of ice formation on the lake, and then a sudden drop to near 0. To remove the 'dark pixel' values a function was built in MATLAB. To identify affected values, the maximum value in 7 days preceding the first affected image is compared to all following values. All lower values are then removed. If all values in the 7-day window are missing, the open water reflectance 390 calculated previously is set as the lower limit.

B.1.7 Phenology events extraction

The extraction of lake-ice phenology was done using the TIMESAT software developed at Lund University for analysis of time series data. In this study the MATLAB-based version of the software was used.

Features in lower portion of the reflectance profile, such as start of freeze-up (FUS) and end of break-up (BUE) are more strongly affected by erogenous bright values (cloud, haze, etc.) To overcome this issue they were from fit adapted to lower envelope. The fit to lower envelope was achieved by setting adaptation strength to 0.1 (increasing weights of values bellow the initial function fit).Features in the upper part of reflectance spectrum, freeze-up end (FUE) and break-up start (BUS), are more susceptible to dark artefacts (e.g. shadow). Therefore, fit to upper envelope was used to extract these events (adaptation strength set to maximum - 10). Due to low sun illumination, FUE is not a distinct feature in most profiles. The best position (40% amplitude) was, as for BUE, judged by visual comparison with *in-situ* data for three reference lakes. For BUS the best position was estimated at 80 % of amplitude.

C. Validation

To assess the accuracy of extracted phenology the results were compared with *in-situ* data. Ground observation data for period 2000 to 2013 were available only for the timing of freeze-up end (FUE) and break-up end (BUE) for selected lakes in north European study area. In total *in-situ* data for 25 lakes were available, resulting in 287 *in-situ* observations for BUE and 274 for FUE. *In-situ* data for other study areas and for freeze-up start (FUS) and break-up start (BUS) were not available.

The extracted dates for BUE showed good agreement with the corresponding *in-situ* observations with mean absolute error (MAE) of 4.7 days and root mean squared error (RMSE) of 6.16 days (Fig. S5). BUE occurs in the period from the end of April to mid-June, when the sun is high in the sky and incoming radiation is high. The BUE is defined as the first day when the lake water is completely free of ice; however, it is not uncommon for lakes to repeatedly break up and refreeze in the course of spring, which complicates the definition of dates.

The results for FUE are not encouraging; the coefficient of determination is 0.32. The mean absolute error equals 24.0 days and RMSE is 32.8 days (**Error! Reference source not found.** S6). This is mostly explained by the fact that FUE generally corresponds with low sun illumination and therefore surface reflectance values for the period of freeze-up are not available, as was discussed previously. The extracted values are consistently overestimated by approximately 17 days. This is most likely the combined effect of data interpolation and the extraction method. As discussed in previous section, the accuracy of the extraction method used (40% amplitude) is determined by the value of the last available observation of surface reflectance. To assess if errors are lake-specific, the extracted variation within time series was analysed. The median standard deviation within the FUE time series is 31 days, as opposed to 7.5 days for BUE. The high standard deviation of FUE values suggests that there is no lake specific bias in the extraction method.

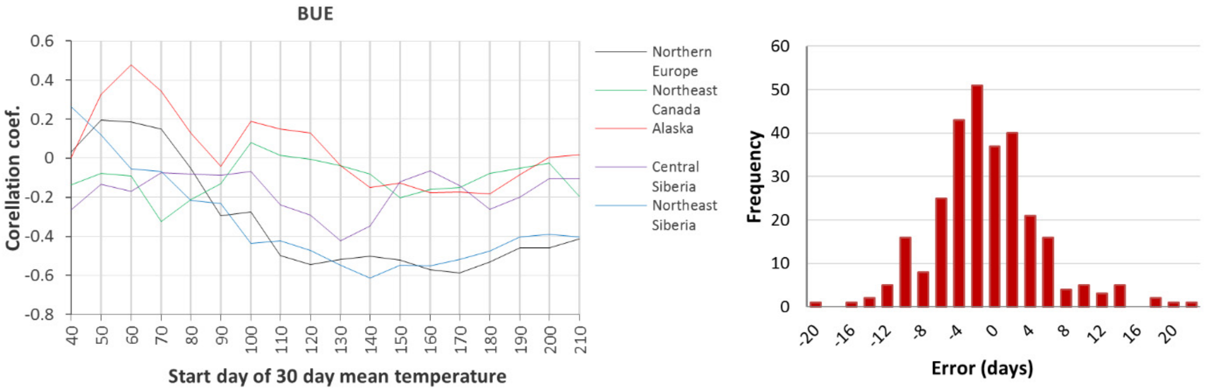

Figure S5 | Comparison of the dates for end of break-up observed in-situ and extracted (left). Absolute error distribution (right). The black line corresponds to a linear trend-line and the dashed line represents the 1:1 relationship. This figure was drawn using MS Excel 2010 (http://www.microsoftstore.com).

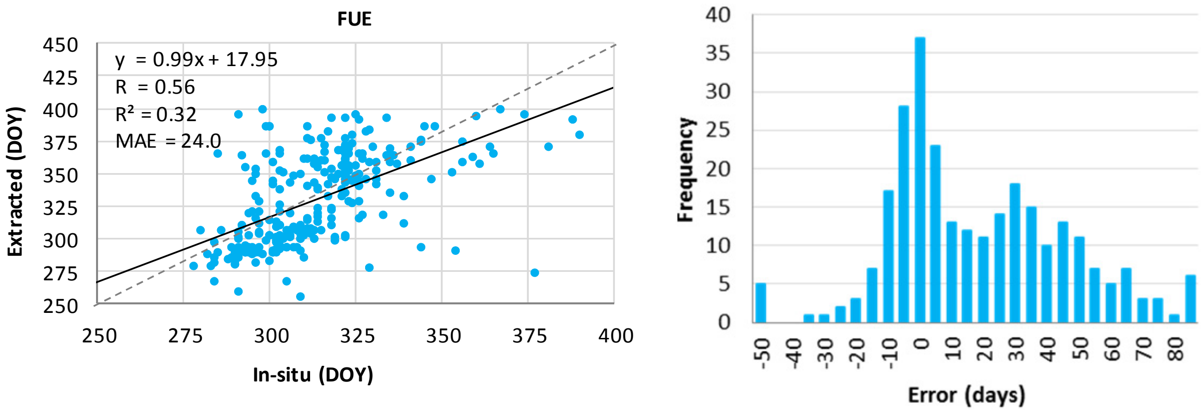

Figure S6 | Comparison of the end of freeze-up dates observed in-situ and extracted (left). Absolute error distribution (right). The black line corresponds to a linear trend-line and the dashed line represents a 1:1 relationship. This figure was drawn using MS Excel 2010 (http://www.microsoftstore.com).

Not all uncertainty can be attributed to the extracted values; a small part can be most likely attributed to the *in-situ* observations. Arctic lake ice often breaks and refreezes several times during course of fall and spring, and multiple dates are, therefore, recorded for FUE and BUE. The last dates are considered as the true timing of complete freeze over and break-up. *In-situ* observations are generally point-based, and often it is not possible to see the entire lake from a single viewpoint.

For extracted dates of freeze-up start and break-up start the variation within time series was also assessed. For BUS the median standard deviation is 7.6 days (in agreement with BUE). For FUS (19.0 days) it is lower than for FUE but still higher than expected. Due to low confidence given to extracted dates for both freeze-up events, further analysis was performed only for the start and end of break-up period.

D. Air Temperature

The final objective of this research was to determine the degree to which temperature determines the timing of BUS and BUE and whether its influence is different among different study areas. As stated previously, due to low confidence given to the extracted timing of freeze-up start and end, only break-up start and break-up end are discussed here. Tested variables are listed in Table S1.

Group	Variable	Definition
Amplitude	Amp.	Annual temperature amplitude (temp. profile smoothed in 31-day window)
0° isotherm	0is	0° C isotherm timing (temp. profile smoothed in 31-day window)
Mean Temperature	T1 – T18	Mean temperature over 30 days in sliding window with 10-day step (40 - 210)

Table S1 | Air temperature and other climatic variables for which the effect on BUS and BUE timing was tested.

In agreement with the literature, the timing of the spring 0°C isotherm, as representative of the annual temperature pattern, explained up to 81% of the variance in BUS and 76% in BUE. The mean sliding window temperatures showed good correlation but only in the northeast Siberian and northern European areas. In other locations the relationship is less clear.

The break-up start in the northeast Siberian and northern European areas strongly correlates with mean temperature for a 30-day period approximately centred in the median BUS date. (Fig. S7) However, in the other study areas the correlation does not increase with time as expected, and it does not follow any recognizable pattern. This is mostly due in inhomogeneity within the study areas.

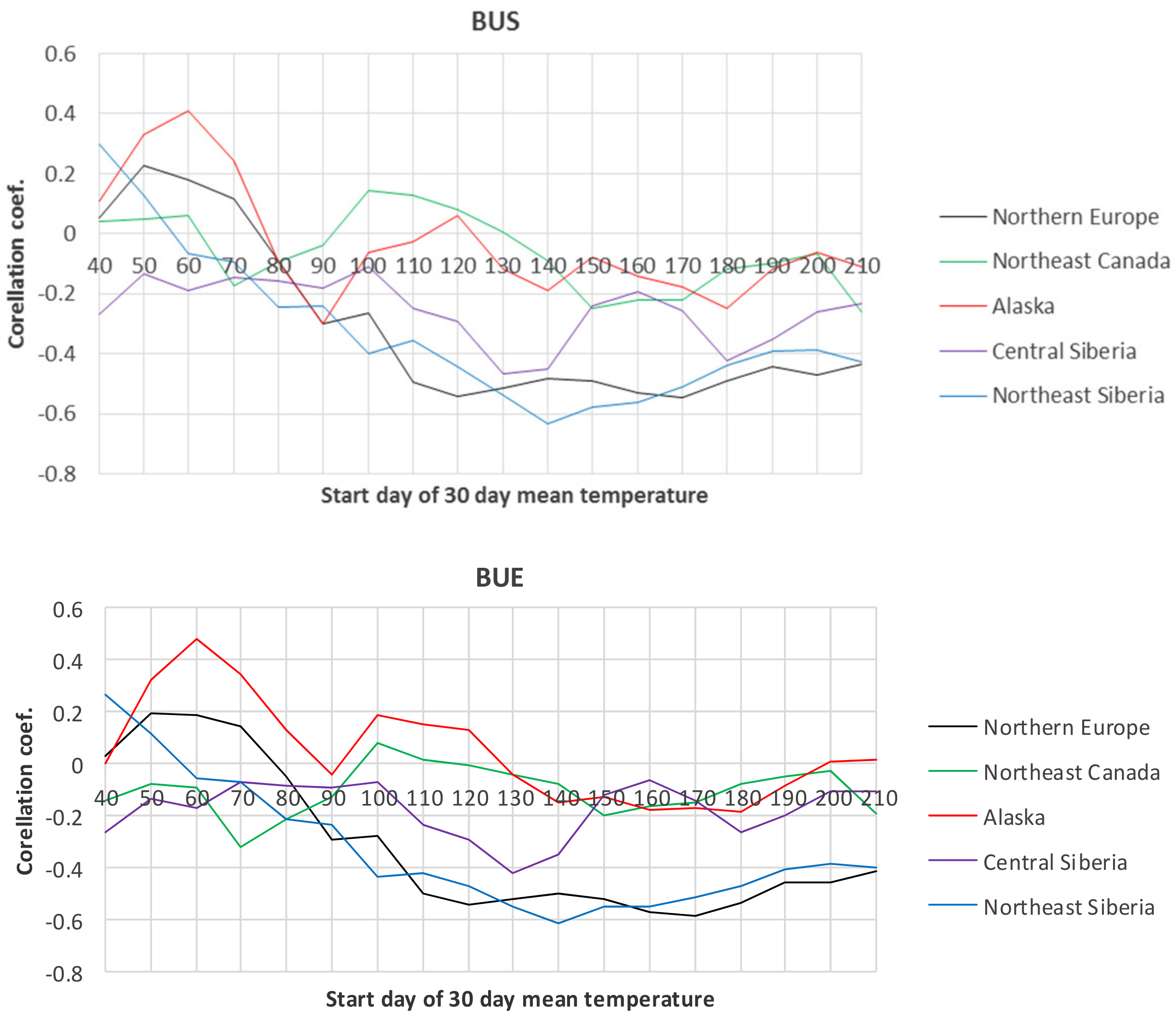

Fig. S7 | Correlation coefficient BUS (top)/BUE (bottom) and mean temperature in 30-day sliding window. This figure was drawn using MS Excel 2010 (http://www.microsoftstore.com).

E. References

1. Vermote, E. F., Kotchenova, S. Y. & Ray, J. P. MODIS Surface Reflectance User's Guide. (2011). at <http://modis-sr.ltdri.org/products.html>
2. Latifovic, R. & Pouliot, D. Analysis of climate change impacts on lake ice phenology in Canada using the historical satellite data record. *Remote Sens. Environ.* **106,** 492–507 (2007).
3. Lehner, B. & Döll, P. Development and validation of a global database of lakes, reservoirs and wetlands. *J. Hydrol.* **296,** 1–22 (2004).
4. Paltan, H., Dash, J. & Edwards, M. A refined mapping of Arctic lakes using Landsat imagery. *Int. J. Remote Sens.* **36,** 5970–5982 (2015).
5. Leys, C., Ley, C., Klein, O., Bernard, P. & Licata, L. Detecting outliers: Do not use standard deviation around the mean, use absolute deviation around the median. *J. Exp. Soc. Psychol.* **49,** 764–766 (2013).
